# Indigenous Medicinal Plants as Biofilm Inhibitors for the Mitigation of Antimicrobial Resistance

**DOI:** 10.1155/2020/8821905

**Published:** 2020-10-24

**Authors:** Elikplim Kwesi Ampofo, Isaac Kingsley Amponsah, Evelyn Asante-Kwatia, Francis Ackah Armah, Philip Kobla Atchoglo, Abraham Yeboah Mensah

**Affiliations:** ^1^Department of Pharmacognosy, Faculty of Pharmacy and Pharmaceutical Sciences, College of Health Sciences, Kwame Nkrumah University of Science and Technology, Kumasi, Ghana; ^2^Department of Biomedical Sciences, School of Allied Health Sciences, University of Cape Coast, Cape Coast, Ghana

## Abstract

The majority of indigenes in the rural areas of Ghana use herbal medicines for their primary health care. In this study, an ethnobotanical survey was undertaken to document medicinal plants used by traditional healers in the Ejisu-Juaben district in the Ashanti region of Ghana to treat infections and to further investigate the antibiofilm formation properties of selected plants in resisting pathogenic bacteria. Seventy medicinal plants used by traditional practitioners for the treatment of skin infections and wounds were documented from the ethnobotanical survey. Forty out of the seventy plants were collected and their methanol extracts evaluated for antimicrobial activity by the agar diffusion assay. Extracts that showed antibacterial activity were tested for biofilm inhibitory activity, and the most active plant was subsequently purified to obtain the active constituents. Biofilm formation was significantly mitigated by petroleum ether, ethyl acetate, and methanol extracts of *Holarrhena floribunda* stem bark. Bioassay-guided fractionation of an alkaloidal extract prepared from the methanol fraction led to the isolation of three steroidal alkaloids, namely, holonamine, holadienine, and conessine. The isolated compounds demonstrated varying degrees of biofilm formation inhibitory properties. The current study reveals that screening of indigenous medicinal plants could unravel potential leads to salvage the declining efficacy of conventional antibiotics. *Holarrhena floribunda* stem bark extract has strong biofilm formation inhibition properties, which could be attributed to the presence of steroidal alkaloids.

## 1. Introduction

Antimicrobial resistance (AMR) has become a major public health concern worldwide as it reduces the effectiveness of antibiotic therapies and increases morbidity, mortality, and health care costs [1]. Current evidence suggests that free-floating bacteria attach onto solid surfaces and create a complex polysaccharide matrix called the biofilm, which protects them from antimicrobial agents [2,3]. Biofilms have great significance for public health because biofilm-associated microorganisms exhibit dramatically high resistance to antimicrobial agents than planktonic forms [4]. This is because in the biofilm state multiple bacterial species form a polymicrobial community with several advantages including a more efficient DNA sharing and quorum sensing system, passive resistance, and metabolic cooperation, which protects them from antimicrobial agents and host immune responses [5].

Some extracts of neotropical rainforest plants have shown remarkable biofilm inhibitory effects [1,6]. Triterpenes with inhibitory effects against *P. aeruginosa* biofilms were isolated from *Diospyros dendo* [7]. The antibiofilm activity of *Azadirachta indica* extract was employed to mitigate microbiologically influenced corrosion in underground pipe lines as an environmentally benign way of managing corrosion [8]. This put natural products with antibiofilm activity in the spotlight for a wide range of applications to address health and industrial issues. In the rural and peri-urban areas like the Ejisu-Juaben district in the Ashanti region of Ghana, access to medical care is limited, and most indigenes rely on herbal medicine practitioners for their primary health needs. Herbal medicines are highly valued and enjoy patronage by a large number of the populace due to its acceptability, affordability, and perceived safety [9]. In this study, an ethnobotanical survey was undertaken in the Ejisu-Juaben district to obtain comprehensive data on plant-based remedies commonly used by traditional healers to treat infection. Furthermore, the biofilm formation inhibitory activities of selected plant extracts were investigated and some bioactive constituents identified.

## 2. Materials and Methods

### 2.1. Chemicals

Chemicals used in the assay were purchased from Sigma and Aldrich chemicals, UK, and Ciprofloxacin was purchased from DENK PHARMA GmbH & Co, Germany. All organic solvents were of analytical grade and obtained from BDH, Laboratory Supplies (Merck Ltd, Lutterworth, UK).

### 2.2. Study Area

**Figure 1:**
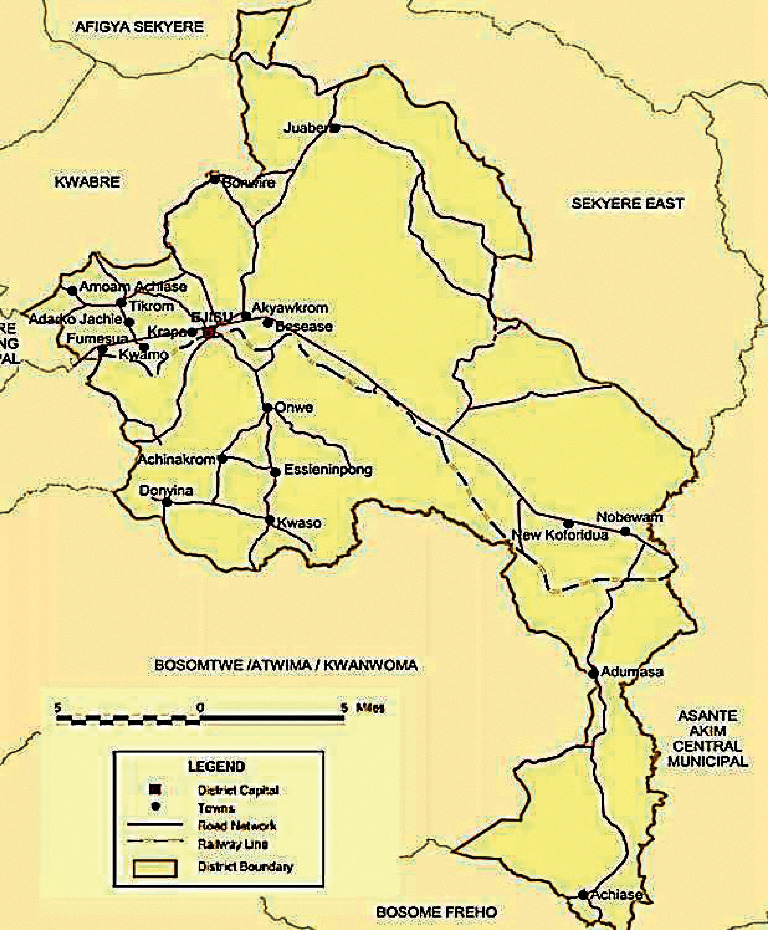
Map of Ejisu-Juaben district (Source: Ghana Statistical Service).

This ethnobotanical study was conducted in the Ashanti Region of Ghana, specifically in the Ejisu-Juaben district (Figure 1). The Ejisu-Juaben district is one of the 27 administrative and political districts in the Ashanti Region of Ghana. It is located in the central part of the region (1.15°N and 1.45°N and longitude 6.15°W and 7.00°W) and has an estimated population of about 150,000 with about 72% residing in the rural areas (Ghana Statistical Service, 2014). The district lies within a semi-deciduous forest zone where flora and fauna is diverse and composed of different species of both economic and ornamental importance. The communities are well known for their traditional beliefs and the use of plants for medicine (Ghana Statistical Service, 2014).

### 2.3. Data Collection

The ethnobotanical survey was carried out in May, 2016, with the help of some members of the Ghana National Association of Traditional Healers, Ashanti region (GNATH), a subunit of the Ghana federation of traditional healers associations (GHAFTRAM). The research was conducted in five villages in the district, namely, Bonwire, Ejisu, Onwe, Besease, and Juaben. Participants were informed of the objectives of the survey and personal visits were made to their homes and practice sites. A prior informed consent form (translated into the local dialect) was given to the participants to sign after the objectives of the study had been explained. In keeping with the traditional customs, appropriate gifts and drinks were given to the participants for the time and the courtesies extended to us. Data were collected through a survey employing open-ended, semi-structured questionnaires in English and translated into the local dialect (Ashanti-Twi). Thirty traditional healers participated in the survey. All participants provided information on the methods of practice, vernacular names of anti-infective plant remedies employed by them, methods of preparation, and administration of these remedies. Ethical approval for the study was obtained from the chiefs of the studied areas and the GNATH.

### 2.4. Plant Material Collection

The interviews were followed by the collection of plants under the supervision of three traditional healers. Seventy medicinal plants were documented from the survey (Supplementary Material-Table S1). Forty of these, cited more than five times by 20% of the traditional healers, were collected from the Ejisu-Juaben district for further investigation. They were authenticated by Dr. George Henry Sam of the Department of Herbal Medicine. Voucher specimens of all the plants have been deposited in the Herbarium of the Herbal Medicine Department, Faculty of Pharmacy & Pharmaceutical Sciences, KNUST, with defined ID numbers. The names and the authenticity of the plant species were confirmed using the Electronic Plant Information Centre (ePIC, http://epic.kew.org/).

#### 2.4.1. Preparation of Plant Extracts for Antibacterial Susceptibility Test

One hundred and fifty grams each of the forty selected plants were dried in the shade for 7 days, milled into a coarse powder using a warring blender, and extracted with 80% methanol by cold maceration. They were filtered, concentrated on a rotary evaporator, and dried on a water bath. They were then stored in a cool dry place until required for use.

#### 2.4.2. Fractionation of *H. floribunda* Extract and Preparation of Alkaloidal Fraction

Coarsely powdered stem bark of *H. floribunda* (3.49 kg) was Soxhlet-extracted successively using 3 L each of petroleum ether (pet-ether), ethyl acetate (EtOAc), and methanol (MeOH) for 48 hours. The extracts obtained were concentrated on a rotary evaporator under low temperature and further dried to afford the pet-ether (PHE-5.2%), ethyl acetate extract (EHE-19.85% w/w), and a methanol extract (MHE-16.15% w/w). An alkaloidal fraction was obtained by dissolving 156.9 g of MHE in 1.25 L of 10% acetic acid. The resulting solution was made distinctly alkaline with 70 mL of strong ammonia. The alkaloids were then extracted with 2.5 L of chloroform. The chloroform extract was concentrated by evaporation on a rotary evaporator to yield 52.4 g of crude alkaloidal fraction.

### 2.5. Antimicrobial Assay

#### 2.5.1. Microorganisms

Resistant clinical strains of *Staphylococcus aureus* ATCC 25923 (A), *Streptococcus pyogenes* clinical strain (B), *Escherichia coli* ATCC 25922 (C), *Pseudomonas aeruginosa* ATCC 27853 (D), and *Klebsiella pneumonia* clinical strain (E) were obtained from the Department of Pharmaceutical Microbiology, Faculty of Pharmacy and Pharmaceutical Sciences, KNUST, and used for the study. They were authenticated according to their respective features on selective media, maintained in 30% glycerol broth and stored at −40°C in a frost-free freezer. The strains were aseptically subcultured into sterile Mueller Hinton broth and incubated at 37° C for 24 hours prior to use. For all assays, the working culture was prepared by standard dilution in distilled water and the bacterial suspension was adjusted to a turbidity matching that of 0.5 McFarland Standard (containing ∼10^8^ colony forming units (CFU)/mL) according to the CLSI guidelines [10,11]. To ensure cellular quiescence, this dilution was maintained at 4°C.

#### 2.5.2. Antimicrobial Susceptibility Test

The agar well diffusion method as described by Jorgenson et al. was used, and the method description partly reproduces their wording [12]. Forty medicinal plants cited more than five times by 20% of the traditional healers (Percentage of respondents with the knowledge about the use of a particular plant) were investigated for antibacterial activity (shown by asterisk ‘^*∗*^'on Table S1, Supplementary Material). Briefly, sterile molten Mueller Hinton agar (20 mL each) was seeded with 100 *μ*L of standardized test organism and poured into 90 mm sterile Petri dishes. After solidification, equivalent wells were drilled in each of the petri dishes using a sterile cork borer (No. 5) and filled with the extracts at concentrations of 5 mg/mL. The petri dishes were allowed to stand for 1 hour at 25°C to allow for diffusion of extracts into the seeded agar and then incubated at 37°C for 24 hours. Zones of growth inhibition were determined after incubation. Ciprofloxacin was used as the reference drug. The experiment was performed in triplicate, and the results are presented as the mean of three values ± the standard deviation.

#### 2.5.3. Analysis of Biofilm Production by Test Organisms

To qualitatively investigate the biofilm-producing abilities of the selected bacteria, the method described by Amaral et al. was employed with some modification. Briefly, a sterile glass slide placed in a sterile petri dish was overlaid with 20 mL of Mueller Hinton broth seeded with 100 *μ*L of standardized culture (∼10^8^ CFU/mL). After 24 and 48 hours of incubation, the slides were washed with normal saline to remove the floating planktonic bacteria, and the biofilm was fixed by heating the slide. Slides were then stained with 0.1% crystal violet for 15 minutes, washed with distilled water, air dried, and observed under the microscope. Photomicrographs of bacterial biofilms were recorded at a magnification of ×100 [13].

#### 2.5.4. Quantitative Determination of the Biofilm Formation by the Organisms

Quantitative assessment of biofilm formation was done using the Crystal Violet (CV) assay as described by Sasirekha et al. and the description partly reproduces their wording [6]. Briefly, fresh Mueller Hinton broth (100 *μ*L) was seeded with 10 *μ*L of standardized microorganism culture in a 96-well microtiter plate and incubated at 37°C for 24 hours. After incubation, the planktonic cells were aspirated and the wells were washed with sterile water to remove the free-floating bacteria. The adhering biofilm was fixed by heating and then stained with 0.1% crystal violet. The wells were then washed with sterile water and dried. The amount of biofilm formed was quantified by adding 200 *μ*L of 95% ethanol/acetic acid mixture (1:1) and the optical density (OD) of the resulting solution was measured at 595 nm. Background absorbance was compensated for by subtracting the optical density (OD) of sterile broth (negative control). The experiment was carried out in triplicate and the mean OD was considered. A cutoff OD was determined from the negative control and used to separate nonbiofilm from biofilm-producing microorganisms following standard methods [11].

#### 2.5.5. Biofilm Inhibition Activity

Eight plant extracts that showed antimicrobial activity in the susceptibility test were investigated in this assay. The test was carried out in 96-well microtiter plates. Briefly, 100 *μ*L of sterile liquid broth medium was inoculated with 10 *μ*L of standardized overnight culture of the selected bacterium, and volumes of plant extracts reconstituted in distilled water were added to obtain concentrations of 10, 5, 2.5, and 1.25 mg/mL. For the determination of the minimum biofilm inhibitory concentrations of fractions and pure compounds, a concentration range between 1 and 0.0078 mg/mL was used. Ciprofloxacin was used as the reference drug. Plates were incubated at 37°C for 24 hours. The experiment was performed in triplicate. After the incubation, the liquid suspension was removed and the amount of biofilm formed determined as described in Section 2.5.4.

### 2.6. Data Management and Analysis

The survey data were analyzed using the Statistical Package for Social Sciences (SPSS) version 22.0 for windows and Graph Pad Prism (Version 5 for windows, San Diego, USA).

### 2.7. Chromatographic Fractionation of Alkaloid Extract

The alkaloid extract (50 g) was loaded onto a glass column (60 cm × 3 cm) packed with silica gel (70–230 mesh). It was then eluted with gradient mixtures of pet-ether, EtOAc, and MeOH. Fifty-six fractions were collected in 60 mL aliquots and bulked together into six subfractions based on their TLC profiles coded CF 1–6. Fraction CF1 (9.5 g) was further purified by column chromatography to obtain sixty-three fractions bulked into six subfractions, which were labeled HF1, HF2, HF3, HF4, HF5, and HF6. Fraction HF1 (1.19 g) was subjected to purification by preparative HPLC [MeOH−H_2_O + 0.1% HCOOH 98 : 2, 2 mL min^−1^] to yield pure compounds, namely, HF1A, HF1B, and HF1C. Details of HPLC experimental procedures are provided in supplementary data (available here).

## 3. Results

### 3.1. Sociodemographic Details of Respondents

All the traditional healers interviewed belonged to the Akan tribe. The inclusion criterion for the survey was that the practitioner used herbal medicine. They comprised 43% men and 57% women aged between 21 and 80 years. The categories of practitioners included diviners (17%), herbalists (67%), clairvoyants (3%), fetish priests (10%), and bone setters (3%). A majority of respondents (61%) had practiced for more than 15 years. Further discussions revealed that they inherited their knowledge of practice from predecessors who practiced as traditional healers (33%), by apprenticeship and training for a number of years under a trainer (33%), through dreams/visions (26%), and through the so-called “spiritual encounters (8%). A majority of respondents (77%) indicated that they harvested their plants from the wild (including collections from a nearby arboretum in the district, Bobiri Forest Reserve) and the rest they cultivated in their farms and backyards (23%).

### 3.2. Plants Used for the Treatment of Infections in the Ejisu-Juaben District

Seventy plants used by the traditional practitioners for the treatment of skin, wounds, and other infections were documented from the survey (Supplementary Material-Table S1). The plants were distributed across 63 genera belonging to 31 families, mostly Fabaceae (9), Euphorbiaceae (6), Moraceae (6), and Meliaceae (6). The vernacular names of plants, uses, methods of preparation, and the administration of remedies were documented.

### 3.3. Antimicrobial Activity of Plant Extracts

**Figure 2:**
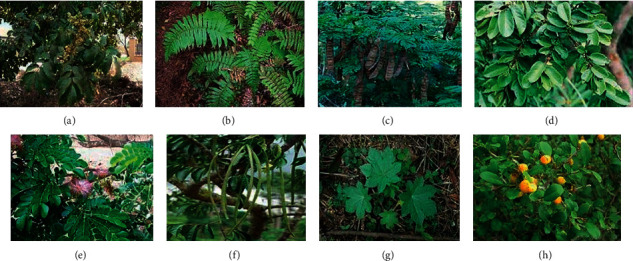
: Selected plants for biofilm formation inhibitory activity. (a) *Trichilia lanata* (MTL). (b) *Grossera vignei* (MGV). (c) *Acacia ataxacantha* (MAA). (d) *Bridelia stenocarpa* (MBS). (e) *Albizia ferruginea* (MAF). (f) *Holarrhena floribunda* (MHF). (g) *Triplochiton scleroxylon* (MTS). (h) *Anogeissus sericea* (MAS).

Forty medicinal plants cited more than five times by 20% of the traditional healers were investigated for antibacterial activity by the agar diffusion assay. Eight out of the forty plants exhibited antimicrobial activities as determined by the zones of inhibition of susceptible microbes (Table 1). These were *Bridelia stenocarpa* (**MBS)**, *Anogeissus sericea* (**MAS)**, *Trichilia lanata* (**MTL**), *Grossera vignei* (**MGV**), *Acacia ataxacantha* (**MAA**)*, Albizia ferruginea* (**MAF**), *Holarrhena floribunda* (**MHF**), and *Triplochiton scleroxylon* (**MTS**) (Figure 2).

### 3.4. Biofilm Formation Ability of Selected Microorganisms

**Figure 3:**
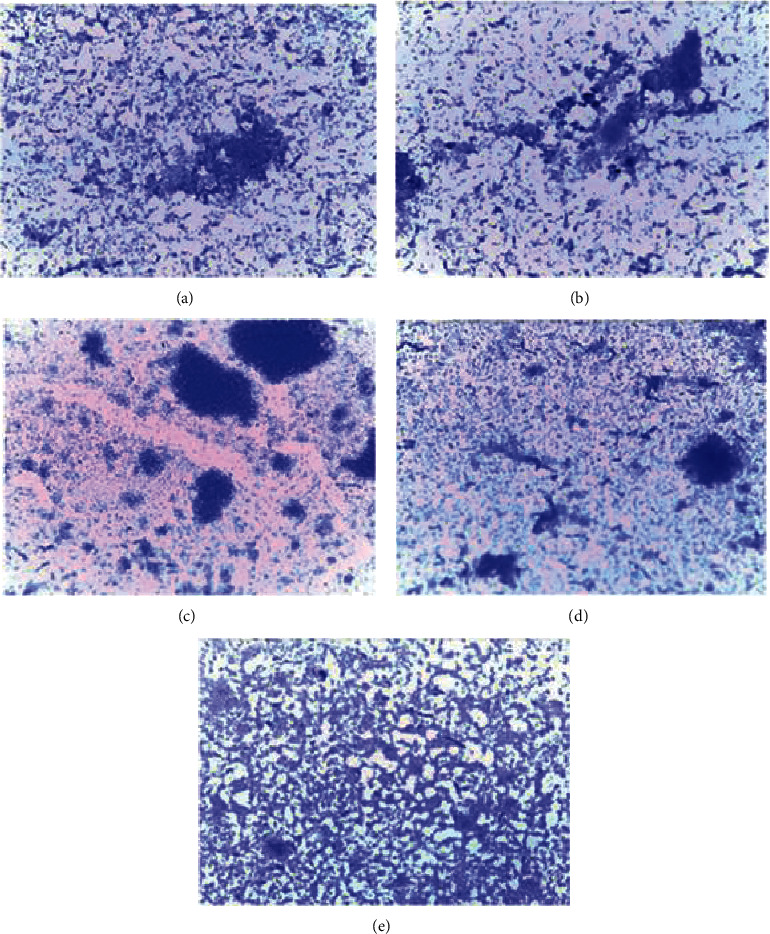
Photomicrographs of adhering bacterial cells (biofilms) stained with 0.1% crystal violet on a glass slide after 24 hours, observed at ×100: (a) *S. aureus*. (b) *S. pyogenes*. (c) *E. coli*. (d) *P. aeruginosa*. (e) *K. pneumoniae*.

All the clinical strains selected for the assay had the ability to produce biofilms. This was first determined qualitatively from photomicrographs of glass slides with adhering bacterial biofilms stained with 0.1% crystal violet (Figure 3). To quantify the amount of the biofilm produced, the optical densities (OD) of reconstituted biofilm solutions from the microtiter plate assay were determined (Table 2). A negative control was included and used to calculate a cutoff OD value, which separates a nonbiofilm producer from a biofilm-producing organism according to the established standards [11,14]. From calculations, the following parameters were set: OD < 0.12 as weak biofilm producers; OD between 0.12 and 0.24 as moderate biofilm producers; and OD > 0.24 as high biofilm producers. The rank order of biofilm-producing ability was *S. aureus > S. pyogenes > E. coli > P. aeruginosa > K. pneumoniae*. *S. aureus* was therefore selected for the investigation of biofilm formation inhibitory property of *H. floribunda.*

### 3.5. Biofilm Formation Inhibitory Effect of Plant Extracts

**Figure 4:**
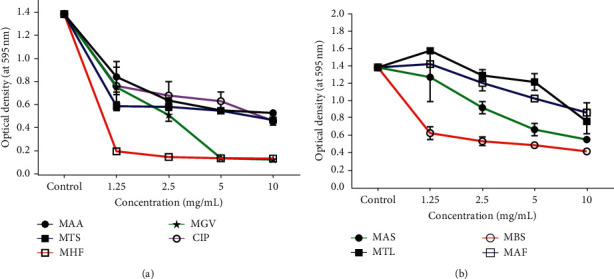
(a-b) Graphs showing the effect of different concentrations of extracts on the amount of biofilm formed (optical density-OD) by *S. aureus*. MBS, *Bridelia stenocarpa*; MAS*, Anogeissus sericea*; MTL, *Trichilia lanata*; MGV, *Grossera vignei*; MAA, *Acacia ataxacantha*; MAF, *Albizia ferruginea*; MHF, *Holarrhena floribunda*; MTS, *Triplochiton scleroxylon;* CIP, ciprofloxacin.

The methanol extracts of the eight plants that showed antibacterial activity were further tested for biofilm inhibitory activity against *S. aureus*. The ability of the extracts to considerably inhibit biofilm formation by a strong biofilm producer (i.e., *S. aureus*) was determined and the minimum biofilm inhibitory concentration (MBIC) recorded. From the experiments, six out of the eight plant extracts failed to significantly inhibit biofilm formation by *S. aureus* at concentrations up to 10 mg/mL (Figure 4(a)–4(b)). Meanwhile, the methanol extracts of two plants, *Grossera vignei* (MGV) and *Holarrhena floribunda* (MHF), showed appreciable biofilm formation inhibitory activities. At a concentration of 1.25 mg/mL, MHF reduced *S. aureus* from a high biofilm producer (OD > 1.38) to a low biofilm-producing organism with an OD < 0.24 (Figure 4(b)). *G. vignei*, methanol extract (MGV) on the other hand, reduced the biofilm production of *S. aureus* to a moderate biofilm producer at concentrations of 5 to 10 mg/mL (Figure 4(b)).

### 3.6. Biofilm Formation Inhibitory Effect of *H. floribunda* Solvent Fractions and Pure Compounds

**Figure 5:**
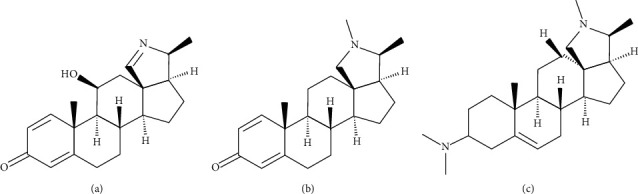
Steroidal alkaloids from the stem bark of *H. floribunda.* (a) Holonamine. (b) Holadienine. (c) Conessine.

**Figure 6:**
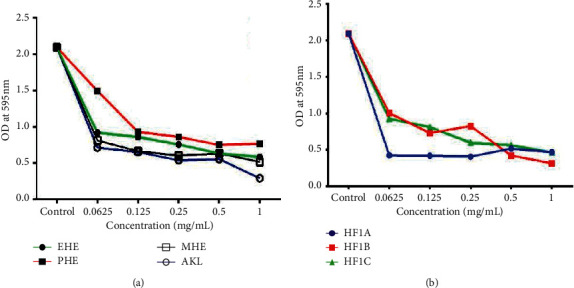
6 (a-b) Graphs showing the effect of different concentrations of fractions and isolated compounds on the amount of biofilm formed (optical density-OD) by *S*. *aureus*. EHE : EtOAc fraction; PHE: pet-ether fraction; MHE : MeOH fraction; AKL: alkaloidal fraction; HF1A: holonamine; HF1B: holadienine; HF1C: conessine.

*H. floribunda* crude extract (MHF), which showed the highest biofilm formation inhibition toward *S. aureus* (Figure 4(a)), was successively fractionated to obtain the pet-ether (PHE), ethyl acetate (EHE), and methanol (MHE) fractions, and their biofilm inhibitory effects were investigated. MHE showed the highest biofilm formation inhibitory effect with an MBIC of 0.0625 mg/mL (Table 3). An alkaloidal fraction (AKL) was further prepared from MHE and purified by column and high-performance liquid chromatography to yield the compounds HF1A, HF1B, and HF1C (Figure 5). The amount of biofilm formed recorded as optical density in the presence of different concentrations of PHE, EHE, MHE, AKL and the isolated compounds toward *S. aureus* is demonstrated in Figure 6(a)–6(b). Among the isolated compounds, HF1A had the highest activity with an MBIC of 0.25 mg/mL toward *S. aureus.*

### 3.7. Characterization of the Compounds

Compounds HF1A, HF1B, and HF1C (Figure 6) were identified based on comparison of mass spectral, ^1^H and ^13^C NMR data to published literature as holonamine, holadienine, and conessine, respectively [15,16].

## 4. Discussion

A number of researches have documented the utilization of herbal medicines in some Ghanaian communities. One of these communities is based in the Ejisu-Juaben district where the use of herbal medicine is widespread and highly diverse due to the floristic and cultural diversity of various communities [17]. In this study, medicinal plants traditionally used for the treatment of infections in the Ejisu-Juaben district have been documented through an ethnobotanical survey. Documentation of such useful medicinal plants is important to facilitate future research on the efficacy of these plants and allow for the appropriate incorporation of the most effective herbal remedies into primary health care.

Further in this study, the biofilm formation inhibitory effect of selected medicinal plants against biofilm-producing *Staphylococcus aureus* was investigated. *S. aureus* biofilms are known to typically colonize both artificial and tissue surfaces in humans and cause serious infections including implant-associated infections, cystic fibrosis, lung infections, and chronic wounds [18]. Among the tested plants, *Holarrhena floribunda* stem bark exhibited the highest biofilm formation inhibitory activity against *S. aureus*. Three extracts, pet-ether (PHE), ethyl acetate (EHE), and methanol (MHE) fractions, were prepared from the crude extract and tested for biofilm inhibitory activity. The methanol fraction exhibited the highest antibiofilm activity among the fractions, extracts, and the reference control. Crude alkaloids (AKL) precipitated from this methanol fraction also showed considerable activity although twofold less active than the methanol fraction (Table 3). Bioassay-guided fractionation of the bioactive alkaloidal fraction revealed the presence of the steroidal alkaloids, holonamine (**HF1A**), holadienine (**HF1B**), and conessine (**HF1C**), which also showed considerable antibiofilm forming activity. These compounds have been previously reported from *H. floribunda* and other *Holarrhena* species [15,16,19]. This is however the first report of the biofilm formation inhibitory effect of *H. floribunda* and the isolated plant constituents. Thus, fractionation of the stem bark extract of *H. floribunda*, to some extent, afforded much potent antibiofilm methanol fraction, which owed its activity, in part, to its constituent alkaloids.

*Holarrhena floribunda* is well known in African traditional medicine for the treatment of malaria, diarrhea, dysentery, diabetes, skin diseases, swellings, and urinary and sexually transmitted infections [20,21]. The antibacterial activity demonstrated by the crude extract and the solvent fractions of the *H. floribunda* stem bark in the current work is consistent with previous reports on the same plant [22–26]. Steroidal alkaloids are the major types of phytoconstituents identified in *H. floribunda* [16,27,28]. Flavonoids, fatty acid esters, and trichothecenes have also been reported [29–31]. The antibacterial activity of *Holarrhena* species has been attributed to the presence of steroidal alkaloids [22,32–34]. Conessine (HF1C) is a well-established constituent of *H. floribunda* [19]. Although the antibiofilm inhibitory property of *H. floribunda* and its constituents was not previously investigated, a recent study demonstrated the resistance-modifying ability of conessine through the inhibition of multidrug efflux pumps [35–38]. Another study has shown that compounds with efflux pump inhibition may also cause biofilm disruption [39]. This assertion makes the current results of the antibiofilm formation effect of conessine very plausible. The activity of the individual isolated compounds was lower than the crude alkaloid fraction, suggesting the possible role of synergism in the activity of the steroidal alkaloids. This is consistent with a previous study by Li-Na et al. [40], which reported synergistic antibacterial activity of steroidal alkaloids isolated from *Holarrhena antidysenterica* against methicillin-resistant *Staphylococcus aureus*. In a previous study, the hydroalcoholic extract *H. antidysenterica* inhibited the formation of biofilm in opportunistic pathogenic *Salmonella typhimurium* [41]. This puts *Holarrhena* species in the spotlight as potential prospects of antibacterial agents with biofilm inhibitory activity.

## 5. Conclusion

This work has provided comprehensive data on the medicinal plants used in the treatment of infection in Ghana. These data can serve as evidential support for the clinical development of a number of medicinal plant remedies as adjuvant therapy. The biofilm formation inhibitory effect of *Holarrhena floribunda* and its constituents validates the ethnomedicinal use of the plant in infectious disease treatment and points to the possible presence of other potentially effective antibacterial constituents in the plant.

## Figures and Tables

**Table 1:** Mean zones of inhibition of extracts in agar well diffusion assay.

Microorganism	Mean zones of inhibition (mm) ± SD								
	MBS	MAS	MTL	MGV	MAA	MAF	MHF	MTS	CIP
*S. aureus*	11.7	12.7	11.3	11.7	21.7	12.3	14.7	16.3	26.7
	±0.7	±2.1	±1.8	±1.2	±2.9	±3.4	±2.4	±3.1	±1.7
*K. pneumoniae*	8.3	10.7	5.7	8.3	12.3	8.7	12.3	14.3	28.7
	±0.9	±2.1	±0.6	±2.1	±3.1	±1.1	±1.9	± 1.5	±0.8
*S. pyogenes*	12.7	9.3	12.7	11.7	20.7	10.3	11.3	10.7	18.3
	±1.9	±1.6	±2.3	±0.8	±4.3	±2.6	±2.0	±2.2	±1.7
*E. coli*	13.7	7.7	4.3	16.7	14.7	9.7	15.7	9.3	19.7
	±4.2	±0.5	±0.8	±4.1	±1.8	±4.1	±0.9	±3.3	±0.9
*P. aeruginosa*	12.3	12.7	13.7	14.3	10.3	11.3	16.7	5.7	10.3
	±1.7	±2.8	±0.9	±2.3	±1.2	±3.7	±0.4	±2.2	±0.6

MBS, *Bridelia stenocarpa;* MAS, *Anogeissus sericea*; MTL, *Trichilia lanata*; MGV, *Grossera vignei;* MAA, *Acacia ataxacantha*; MAF, *Albizia ferruginea*; MHF, *Holarrhena floribunda* MTS, *Triplochiton scleroxylon;* CIP, ciprofloxacin; SD, standard deviation.

**Table 2:** Optical densities of biofilms produced by various microorganisms.

*Organism*	*S. aureus*	*S. pyogenes*	*E. coli*	*P. aeruginosa*	*K. pneumoniae*	Control
**OD**	1.38 ± 0.049	1.37 ± 0.083	1.25 ± 0.034	1.15 ± 0.103	0.86 ± 0.044	0.05 ± 0.004

OD, optical density.

**Table 3:** Minimum biofilm inhibitory concentrations of fractions, isolated compounds HF1A, HF1B, and HF1C, and the reference drug against *S. aureus*.

Extracts/compounds	Minimum biofilm inhibitory concentration (MBIC) (mg/mL)
MHF	1.00
PHE	>1.00
MHE	0.0625
EHE	>1.00
AKL	0.125
HF1A	0.250
HF1B	>1.00
HF1C	0.50
CIP	0.50

CIP: ciprofloxacin; MHF: crude extract; MHE: methanol fraction; EHE: ethyl acetate fraction; PHE: pet-ether fraction; AKL: alkaloidal fraction. HF1A, HF1B, and HF1C are isolated compounds.

## Data Availability

The raw data/results from experiments used to arrive at the findings of this study are available from the corresponding author upon request. Previous reports that were used to support this study are cited at relevant places within the text as references.

## Supplementary Materials

Supplementary Materials Methods: general experimental procedures. Liquid chromatography-mass spectrometry (LC-MS). Results: Table S1: medicinal plants used for the management of skin and wound infections as well as other infective conditions in the Ejisu-Juaben district of Ghana.
