# Supplementary material for: Indigenous Medicinal Plants as Biofilm Inhibitors for the Mitigation of Antimicrobial Resistance
Source: Adv Pharmacol Pharm Sci. 2020 Oct 24;2020:8821905. doi: 10.1155/2020/8821905 (PMC7604581; doi:10.1155/2020/8821905)
Supplement: Supplementary Materials — Methods: general experimental procedures. Liquid chromatography-mass spectrometry (LC-MS). Results: Table S1: medicinal plants used for the management of skin and wound infections as well as other infective conditions in the Ejisu-Juaben district of Ghana. [file 8821905.f1.docx]

**Indigenous medicinal plants as biofilm inhibitors for the mitigation of antimicrobial resistance**

Elikplim Kwesi Ampofo ^1^, Isaac Kingsley Amponsah ^1^, Evelyn Asante-Kwatia ^1*^, Francis Ackah Armah ^2^, Philip Kobla Atchoglo ^1^, Abraham Yeboah Mensah ^1^

**^1^** Department of Pharmacognosy, Faculty of Pharmacy and Pharmaceutical Sciences, College of Health Sciences, Kwame Nkrumah University of Science and Technology, Kumasi, Ghana.

**^2^** Department of Biomedical Sciences, School of Allied Health Sciences, University of Cape Coast, Cape Coast, Ghana

***Corresponding author:**

Evelyn Asante-Kwatia

Department of Pharmacognosy, Faculty of Pharmacy and Pharmaceutical Sciences, College of Health Sciences, Kwame Nkrumah University of Science and Technology, Kumasi, Ghana. Email: [eamireku@knust.edu.gh](mailto:eamireku@knust.edu.gh); Telephone: +233246471364

**SUPPLEMENTARY MATERIAL**

**Methods**

*2.2 General Experimental Procedures*

Nuclear magnetic resonance (NMR) data were recorded at 25 ˚C on a Bruker Avance DRX-500 (500 MHz) using deuterated chloroform (CDCl_3_) and methanol-*d_4_* (CD_3_OD) as solvents. Column chromatography was performed using silica gel 60 (70−230 mesh; AppliChem, GmbH, Darmstadt, Germany). The HPLC separations were performed using a Nexera Shimadzu LC using a Phenomenex Gemini C18 column (10 x 250, 10 µm particle size) and a chromeleon software system. The set up consisted of a gynkotek pump equipped with a Dionex DG-1210 degasser, a Dionex UVD 340S detector and a Dionex Gina 50 auto-sampler. During the separation, MeOH-H_2_O solvent system was employed for travelling the RP column with 0.1% formic acid (HCOOH) as buffer. The gradient of 95% methanol 5 % water to 100 % water was employed in the run.

*2.2.1 Liquid Chromatography-Mass Spectrometry (LC-MS)*

The HPLC-MS experiments were carried out on a LTQ Orbitrap spectrometer (Thermofisher, USA) equipped with a HESI-II source. The spectrometer is equipped with an Agilent 1200 (Santa Clara, USA) HPLC system consisting of a pump, PDA detector, column oven (30 ˚C) and an auto sampler. It was operated with nominal mass resolving power of 60 000 at *m/z* 400 with a scan rate of I Hz to afford high-accuracy mass measurements within 2 ppm deviation. The HPLC separations were performed using a Luna C18 column 10 x 250 mm, 10 μm particle size (Phenomenex, Torrance, USA) with H_2_O (+ 0.1 % HCOOH) (A) / MeOH (+ 0.1 % HCOOH (B), gradient flow rate 0.4 mL/min. Samples were analysed by using programme as follows; linear gradient from 5 % A to 100 % B over 26 min, 100 % B isocratic for 5 min, then after the system returned to its initial condition (95 % A, 5 % B) within 0.5 min and was equilibrated for 4.5 min. The experiments were run in positive mode. TLC was carried out with pre-coated silica gel 60 plates (0.25 mm; Merck, Darmstadt, Germany). Separated compounds were visualized by spraying with H_2_SO_4_−EtOH (1:9, v/v) followed by heating. All spectroscopic data obtained were compared with published data for known compounds.

**Results**

**Table S1: Medicinal plants used for the management of skin and wound infections as well as other infective conditions in Ejisu-Juaben district of Ghana**

| **Family** | **Scientific Name** | **Voucher Number** | **Local Name** | **Treatment Type** | **Formulation** | **Part Used** |
| --- | --- | --- | --- | --- | --- | --- |
| Anacardiaceae | *Mangifera* *indica* L.  *Spondias* *mombin* Jacq. | HM/M001  HM/S002 | Amango  Afoa | Stomach ulcer  Skin ulcer | Decoction  Poultice, powder | Fresh bark  Dried bark |
| Annonaceae | ******Cleistopholis* *patens* [Engl.](http://www.ipni.org/ipni/idAuthorSearch.do?id=18509-1&back_page=) & [Diels](http://www.ipni.org/ipni/idAuthorSearch.do?id=18030-1&back_page=) | HM/C003 | Nkyenenego | Sores | Decoction | Bark |
| Apocynaceae | *Alstonia* *boonei* [De Wild.](http://www.ipni.org/ipni/idAuthorSearch.do?id=12406-1&back_page=)  ******Funtumia* *elastica* ( [P. Preuss](http://www.ipni.org/ipni/idAuthorSearch.do?id=13687-1&back_page=) ) [Stapf](http://www.ipni.org/ipni/idAuthorSearch.do?id=27136-1&back_page=)  ******Holarrhena* *floribunda* [T. Durand](http://www.ipni.org/ipni/idAuthorSearch.do?id=2400-1&back_page=) & Schinz | HM/A004  HM/F005  HM/H006 | Osinuro  Ofuntum  Osese | Sores  Sores  sores | Paste, poultice  Poultice  Decoction, poultice | Bark exudates  Stem bark  Stem bark |
| Araliaceae | *Cussonia* *bancoensis* [Aubrév.](http://www.ipni.org/ipni/idAuthorSearch.do?id=328-1&back_page=) & [Pellegr.](http://www.ipni.org/ipni/idAuthorSearch.do?id=7474-1&back_page=) | HM/C007 | Kwaeboofre | Skin lessions | Decoction, hot infusion | Stem bark, leaves |
| Asclepiadaceae | ******Gongronema* *latifolium* [Benth.](http://www.ipni.org/ipni/idAuthorSearch.do?id=15956-1&back_page=) | HM/G008 | Nsurogya | Skin infections | Powder, decoction | Leaves, roots |
| Bignoniaceae | *Newbouldia* *laevis* ( [P.Beauv.](http://www.ipni.org/ipni/idAuthorSearch.do?id=7271-1&back_page=) ) [Seem.](http://www.ipni.org/ipni/idAuthorSearch.do?id=9417-1&back_page=)  ******Spathodea* *campanulata* [Buch.-Ham.](http://www.ipni.org/ipni/idAuthorSearch.do?id=1242-1&back_page=) ex [DC.](http://www.ipni.org/ipni/idAuthorSearch.do?id=16855-1&back_page=) | HM/N009  HM/S010 | Asosomasa  Akuakuanuso | Skin infections  Painful lession | Poultice  Poultice | Bark, leaves  Stem bark, roots |
| Bombacaceae | ******Bombax* *buonopozense* [P.Beauv.](http://www.ipni.org/ipni/idAuthorSearch.do?id=7271-1&back_page=)  ******Ceiba* *pentandra* ([L.](http://www.ipni.org/ipni/idAuthorSearch.do?id=12653-1&back_page=)) [Gaertn.](http://www.ipni.org/ipni/idAuthorSearch.do?id=2974-1&back_page=) | HM/B011  HM/C012 | Akata  Onyina | Sores  Ear infections | Powder, decoction  Decoction | Leaves, roots  Roots |
| Boraginaceae | ******Cordia* *millenii* [Baker](http://www.ipni.org/ipni/idAuthorSearch.do?id=407-1&back_page=) | HM/C013 | Kyeneboa akoa | Pimples | Decoction | Stem bark, roots |
| Cecropiaceae | ******Musanga* *cecropioides* R.Br.apud Tedlie  *Myrianthus* *arboreus* [P.Beauv.](http://www.ipni.org/ipni/idAuthorSearch.do?id=7271-1&back_page=) | HM/M014  HM/M015 | Ogyama  Nyakomaanini | Ear infections  Skin rashes | Decoction, hot infusion  Poultice | Leaves, bark  Leaves, bark |
| Celastraceae | *Hippocratea* *africana* [Loes.](http://www.ipni.org/ipni/idAuthorSearch.do?id=5706-1&back_page=) ex [Engl.](http://www.ipni.org/ipni/idAuthorSearch.do?id=18509-1&back_page=)  ******Reissantia* *indica* ([Willd.](http://www.ipni.org/ipni/idAuthorSearch.do?id=11692-1&back_page=)) [N.Hallé](http://www.ipni.org/ipni/idAuthorSearch.do?id=3584-1&back_page=)  ******Salacia* *debilis* [Walp.](http://www.ipni.org/ipni/idAuthorSearch.do?id=11355-1&back_page=) | HM/H016  HM/R017  HM/S018 | Ntwea  Hyera  Ahomakyɛm | Ringworm  Wounds  Ringworm | Poultice, powder  Decoction, poultice  Poultice | Stem bark  Whole stem  Stem bark |
| Clusiaceae | *Garcinia* *kola* [Heckel](http://www.ipni.org/ipni/idAuthorSearch.do;jsessionid=9716454BC2330DDDDEDA9B8EE4E543FA?id=3763-1&back_page=) | HM/G019 | Tweapea | Skin ulcers | Paste, decoction | Stem bark, seed |
| Combretaceae | ******Anogeissus* *sericea* [Brandis](http://www.ipni.org/ipni/idAuthorSearch.do?id=1076-1&back_page=)  *Combretum* *racemosum* [P.Beauv.](http://www.ipni.org/ipni/idAuthorSearch.do?id=7271-1&back_page=) | HM/A020  HM/C021 | Kanɛnini  Ohwerem | Skin infections  Razor bumps | Decoction, poultice  Poultice | Stem bark  Stem, leaves |
| Convolvulaceae | ******Calycobolus* *africanus* ([G.Don](http://www.ipni.org/ipni/idAuthorSearch.do?id=2268-1&back_page=)) [Heine](http://www.ipni.org/ipni/idAuthorSearch.do?id=3791-1&back_page=) | HM/C022 | Kronini | Nose bleed, wounds | Poultice | Stem |
| Crassulaceae | ******Kalanchoe* *integra* [Kuntze](http://www.ipni.org/ipni/idAuthorSearch.do?id=12634-1&back_page=) | HM/K023 | Apuro | Haemostatic | Poultice, decoction | Stem bark |
| Dracaenaceae | *Dracaena* *arborea* Hort.Angl. ex [Link](http://www.ipni.org/ipni/idAuthorSearch.do?id=22401-1&back_page=) | HM/D024 | Ntonmɛ | Ringworm | Poultice | Leaves |
| Euphorbiaceae | *Bridelia* *ferruginea* [Benth.](http://www.ipni.org/ipni/idAuthorSearch.do?id=15956-1&back_page=)  ******Bridelia* *stenocarpa* [Müll.Arg.](http://www.ipni.org/ipni/idAuthorSearch.do?id=23712-1&back_page=)  ******Erythrococca* *africana* [Prain](http://www.ipni.org/ipni/idAuthorSearch.do?id=7925-1&back_page=)  ******Grossera* *vignei* [Hoyle](http://www.ipni.org/ipni/idAuthorSearch.do?id=4171-1&back_page=)  *Margaritaria* *discoidea* ([Baill.](http://www.ipni.org/ipni/idAuthorSearch.do?id=31201-1&back_page=)) [G.L.Webster](http://www.ipni.org/ipni/idAuthorSearch.do?id=11479-1&back_page=)  ******Ricinodendron* *heudelotii*([Baill.](http://www.ipni.org/ipni/idAuthorSearch.do?id=31201-1&back_page=))[Pierre](http://www.ipni.org/ipni/idAuthorSearch.do?id=7689-1&back_page=) ex[Heckel](http://www.ipni.org/ipni/idAuthorSearch.do?id=3763-1&back_page=) | HM/B025  HM/B026  HM/E027  HM/G028  HM/M029  HM/R030 | Opam  Opamfufuo  Oyaanini  Odubrafoo  Pepea  Wanma | Ulcer  Boil  Wounds  Stomach ache  Sores  Stomache ache | Decoction  Poultice  Poultice  Hot infusion  Powder, poultice  Hot infusion | Stem bark  Stem bark  Leaves  Leaves  Stem bark  Fruit |
| Leguminoseae | ******Indigofera* *hirsuta* L  ******Acacia* *ataxacantha* [DC.](http://www.ipni.org/ipni/idAuthorSearch.do?id=16855-1&back_page=)  ******Albizia* *ferruginea* [Benth.](http://www.ipni.org/ipni/idAuthorSearch.do?id=15956-1&back_page=)  ******Albizia* *zygia* [J.F.Macbr.](http://www.ipni.org/ipni/idAuthorSearch.do?id=5889-1&back_page=)  ******Baphia* *nitida* Lodd.  ******Erythrina* *sigmoidea* Hua  *Piptadeniastrum* *africanum* ([Hook.f.](http://www.ipni.org/ipni/idAuthorSearch.do?id=4084-1&back_page=)) [Brenan](http://www.ipni.org/ipni/idAuthorSearch.do?id=1118-1&back_page=)  *Pterocarpus* *erinaceus* [Poir.](http://www.ipni.org/ipni/idAuthorSearch.do?id=24945-1&back_page=)  *Tetrapleura* *tetraptera* [Taub.](http://www.ipni.org/ipni/idAuthorSearch.do?id=10451-1&back_page=) | HM/I031  HM/A032  HM/A033  HM/A034  HM/B035  HM/E036  HM/P037  HM/P038  HM/T039 | Some  Nwerɛ  Opampena  Okoro  Odwen  Tentreni  Dannan  Duakoni  Prɛkɛsɛ | Skin ulcer  Genital ulcer  Wounds  Wounds  Wounds  Skin rashes  Skin rashes  Parasitic infections  Skin eruptions | Poultice  Poultice  Paste/powder  Decoction, poultice  Poultice, decoction  Decoction  Decoction  Powder, decoction  Hot infusion | Whole stem  Stem bark  Stem bark  Stem bark  Stem bark  Stem bark  Stem bark  Stem bark  Fruit |
| Lythraceae | ******Lawsonia* *inermis* L. | HM/L040 | Leele | Wounds | Poultice | Root |
| Meliaceae | *Entandrophragma* *angolense* [C.DC.](http://www.ipni.org/ipni/idAuthorSearch.do?id=2065-1&back_page=)  *Khaya* *senegalensis* [A.Juss.](http://www.ipni.org/ipni/idAuthorSearch.do?id=4593-1&back_page=)  ******Trichilia* *lanata* [A.Chev.](http://www.ipni.org/ipni/idAuthorSearch.do?id=1584-1&back_page=)  *Trichilia* *monadelpha* ([Thonn.](http://www.ipni.org/ipni/idAuthorSearch.do?id=10624-1&back_page=)) [J. J. de Wilde](http://www.ipni.org/ipni/idAuthorSearch.do?id=11675-1&back_page=)  *Turraea* *heterophylla* [Harv.](http://www.ipni.org/ipni/idAuthorSearch.do?id=3705-1&back_page=) & [Sond.](http://www.ipni.org/ipni/idAuthorSearch.do?id=9891-1&back_page=) | HM/E041  HM/K042  HM/T043  HM/T044  HM/T045 | Kokote  Duabini  Tanuronnua  Tanuronini  Ahunu anyankwa | Parasitic disease  Wounds  Wounds  Parasitic diseases  Stomach ulcer | Decoction  Decoction  Poultice, powder  Decoction  Decoction | Stem bark  Stem bark  Stem bark  Stem bark, root  Roots |
| Moraceae | *Antiaris* *toxicaria* ([Pers.](http://www.ipni.org/ipni/idAuthorSearch.do?id=24750-1&back_page=)) [Lesch.](http://www.ipni.org/ipni/idAuthorSearch.do?id=5501-1&back_page=)  *Chlorophora* *regia* [A.Chev.](http://www.ipni.org/ipni/idAuthorSearch.do?id=1584-1&back_page=)  *Ficus* *exasperata* [Vahl](http://www.ipni.org/ipni/idAuthorSearch.do?id=28125-1&back_page=)  ******Ficus* *sur* [Forssk.](http://www.ipni.org/ipni/idAuthorSearch.do?id=12502-1&back_page=)  ******Morus* *mesozygia* [Stapf](http://www.ipni.org/ipni/idAuthorSearch.do?id=27136-1&back_page=)  ******Treculia* *africana* [Decne.](http://www.ipni.org/ipni/idAuthorSearch.do?id=2063-1&back_page=) ex [Trécul](http://www.ipni.org/ipni/idAuthorSearch.do?id=10804-1&back_page=) | HM/A046  HM/C047  HM/F048  HM/F049  HM/M050  HM/T051 | Kyɛnkyɛn  Odum  Oyankyerɛn  Odoma  Odumma  Ofoso | Eczema, wounds  Burns, wounds  Wounds, oedema  Diarrhoea  Dysentry, syphilis  Ear and throat infections | Poultice  Decoction  Poultice  Poultice  Decoction  Ear drop, Gargle decoction | Bark  Stem bark  Leaves, Latex  Stem bark  Stem bark  Stem bark, leaves |
| Myrtaceae | *Psidium* *guajava* L. | HM/P052 | Oguawa | Diarrhoea,dysentry | Decoction | Leaves |
| Oleaceae | ******Jasminum* *dichotomum* [Vahl](http://www.ipni.org/ipni/idAuthorSearch.do?id=28125-1&back_page=) | HM/J053 | Krampa | Skin infection | Poultice, infusion | Leaves |
| Passifloraceae | *Adenia* *cissampeloides* ([Planch.](http://www.ipni.org/ipni/idAuthorSearch.do?id=7738-1&back_page=) ex [Benth.](http://www.ipni.org/ipni/idAuthorSearch.do?id=15956-1&back_page=)) [Harms](http://www.ipni.org/ipni/idAuthorSearch.do?id=3667-1&back_page=)  *Adenia* *lobata* [Engl.](http://www.ipni.org/ipni/idAuthorSearch.do?id=18509-1&back_page=) | HM/A054  HM/A055 | Homa kyɛm  Homa kyɛm | Sores, diabetes  Sores | Decoction  Decoction | Aerial part  Aerial part |
| Polygalaceae | *Securidaca longipedunculata* Fresen | HM/S056 | Afodoo | Ulcer | Infusion | Stem bark |
| Rhamnaceae | ******Gouania* *longipetala* [Hemsl.](http://www.ipni.org/ipni/idAuthorSearch.do?id=3825-1&back_page=) | HM/G057 | Sokuruwa | Wounds | Decoction | Whole stem |
| Rubiaceae | *Hallea* *stipulosa* ([DC.](http://www.ipni.org/ipni/idAuthorSearch.do?id=16855-1&back_page=)) [J.-F. Leroy](http://www.ipni.org/ipni/idAuthorSearch.do?id=5498-1&back_page=)  *Psydrax* *subcordata* ([DC.](http://www.ipni.org/ipni/idAuthorSearch.do?id=16855-1&back_page=)) [Bridson](http://www.ipni.org/ipni/idAuthorSearch.do?id=1139-1&back_page=) | HM/H058  HM/P059 | Subaba  Ogyapam | Dysentry  Wounds | Decoction  Powder | Stem bark  Stem bark |
| Sapindaceae | *Blighia* *sapida* [K. D. Koenig](http://www.ipni.org/ipni/idAuthorSearch.do?id=12620-1&back_page=)  ******Blighia* *unijugata* [Baker](http://www.ipni.org/ipni/idAuthorSearch.do?id=407-1&back_page=)  ******Cardiospermum* *halicacabum* L. | HM/B060  HM/B061  HM/C062 | Akyefufuo  Akyetuntum  Kokoo | Wounds  Ulcers  Snake bite | Decoction  Decoction  Poultice | Stem bark  Stem bark  Leaves |
| Sapotaceae | *Vitellaria* *paradoxa* [C.F.Gaertn.](http://www.ipni.org/ipni/idAuthorSearch.do?id=2973-1&back_page=) | HM/V063 | Krenku | Wounds | Hot infusion | Fruit |
| Smilacaceae | *Smilax* *kraussiana* [Meisn.](http://www.ipni.org/ipni/idAuthorSearch.do?id=12678-1&back_page=) | HM/S064 | Kokora | Skin infections | Poultice | Root |
| Sterculiaceae | *Cola* *nitida* ([Vent.](http://www.ipni.org/ipni/idAuthorSearch.do?id=11128-1&back_page=)) [Schott](http://www.ipni.org/ipni/idAuthorSearch.do?id=9240-1&back_page=) & [Endl.](http://www.ipni.org/ipni/idAuthorSearch.do?id=18490-1&back_page=)  *Theobroma* *cacao* [L.](http://www.ipni.org/ipni/idAuthorSearch.do?id=12653-1&back_page=)  ******Triplochiton* *scleroxylon* [K.Schum.](http://www.ipni.org/ipni/idAuthorSearch.do?id=9333-1&back_page=)  *Sterculia africana* (Lour.) Fiori | HM/C065  HM/T066  HM/T067  HM/T068 | Bese  Kokoo  Owawa  Akwankwa | Skin infection  Wounds  Oedema, sores  Wounds | Hot Infusion  Poultice  Cold infusion  Decoction | Fruit  Fruit  Leaves  Stem bark |
| Ulmaceae | *Trema orientalis* Linn. Blume  *Celtis* *africana* [Burm.f.](http://www.ipni.org/ipni/idAuthorSearch.do?id=12368-1&back_page=) | HM/T069  HM/C070 | Sesea  Ɛsa | Skin diseases  Wounds | Decocotion  Poultice | Stem bark  Stem bark |

Plants marked with asterik (*****) were selected for preliminary microbial susceptibility testing

*Physicochemical properties of isolated compounds:*

*Compound HF1A* (Holonamine): White amorphous powder. Positive ESI-HRMS: *m/z* 326.2021 [M+H]^+^ (calculated for C_21_H_27_NO_2_).

*Compound HF1B* (Holadienine): Yellow amorphous powder. Positive ESI-HRMS: *m/z* 326.2481 [M+H]^+^ (calculated for C_22_H_31_NO).

*Compound HF1C* (Conessine): Yellow crystal; melting point: 125-127 ˚C (literature: 126-127 ˚C). Positive ESI-HRMS: *m/z* 357.3267, [M+H]^+^ (calculated for C_24_H_41_N_2_).
